# Untapped Potential of Recycled Thermoplastic Blends in UD Composites via Finite Element Analysis

**DOI:** 10.3390/polym17091168

**Published:** 2025-04-25

**Authors:** Pei Hao, Ninghan Tang, Juan Miguel Tiscar, Francisco A. Gilabert

**Affiliations:** 1 Department of Materials, Textiles and Chemical Engineering (MaTCh), Mechanics of Materials and Structures (MMS), Tech Lane Ghent Science Park-Campus A, Ghent University (UGent), Technologiepark-Zwijnaarde 46, 9052 Ghent, Belgium; pei.hao@ugent.be (P.H.); ninghan.tang@ugent.be (N.T.); 2 Instituto de Tecnología Cerámica (ITC), Asociación de Investigación de las Industrias Cerámicas (AICE), Universitat Jaume I, Campus Universitario Riu Sec, 12006 Castellón, Spain; juanmiguel.tiscar@itc.uji.es

**Keywords:** recycled thermoplastics, fiber-reinforced plastics, polymer blends, mechanical behavior, micro-mechanical modeling, stress–strain predictions

## Abstract

The increasing demand for fully recyclable composites has spurred extensive research on thermoplastics, valued for their recyclability and excellent mechanical properties. High-performance thermoplastics such as PEEK and PPS have been widely adopted in aerospace applications due to their outstanding load-bearing capabilities, which are well documented. Recently, thermoplastic polymer blends have gained attention for their enhanced recyclability and sustainability, as well as their ability to improve thermal stability, viscosity, and manufacturability. However, limited data are available on the mechanical characterization of composites that incorporate these blends, particularly when recycled thermoplastics are used. In this study, we first examine the stress–strain behavior of the following three polymer blends relevant for structural applications: PES/PEEK, PPS/PEEK, and HDPE/PP. We then perform a numerical analysis to predict the mechanical performance of unidirectional fiber-reinforced composites using each blend as the matrix. This involves a micromechanical Representative Volume Element (RVE) approach combined with an advanced polymer model previously validated against experimental data. The findings are discussed to critically assess the suitability of these blends for producing fully matrix-recycled composites.

## 1. Introduction

The increasing demand for fully recyclable composite materials has driven significant interest in the use of thermoplastics, which offer both recyclability and high-performance characteristics [1,2]. In particular, high-performance thermoplastics have gained prominence in the automotive and aerospace industries due to their excellent mechanical properties, chemical resistance, and thermal stability [3,4,5]. However, the aerospace sector, like many others, faces growing sustainability concerns, necessitating the development of more environmentally friendly material solutions. One emerging approach to enhance the sustainability of these materials is the use of polymer blends, which can improve recyclability while maintaining or enhancing performance [6,7]. Additionally, in the broader context of recyclable polymers, polypropylene (PP) and polyethylene (PE) remain the most widely used recycled commodity thermoplastics [8]. There have also been efforts to develop natural fiber-reinforced thermoplastic composites as a sustainable alternative, combining renewable reinforcement materials with recyclable polymer matrices to further advance circular economy principles in composite manufacturing [9,10,11,12,13].

Despite the growing interest in recyclable thermoplastic composites, several research gaps must be addressed to fully realize their potential. One critical challenge is the lack of comprehensive mechanical characterization data for composites incorporating recycled thermoplastic blends. While these materials are typically tested following ASTM and ISO standards, the resulting datasheet properties provide only a basic understanding of their mechanical behavior [3,13,14,15,16]. This limited characterization makes it difficult to accurately assess key failure mechanisms, such as matrix cracking, fiber breakage, interface debonding, and delamination, which are crucial for ensuring structural integrity and reliability. Tiwari et al. [17] performed SEM analysis of the fracture surfaces, which showed that 50/50 polyphenylene sulfide (PPS)/polyether ether ketone (PEEK) blends were homogeneous, with dispersed sphere-shaped PEEK particles at various milled carbon fiber concentrations and good fiber–matrix adhesion. Although efforts have been made to integrate polymer blends into composite materials, there is still a significant lack of in-depth mechanical performance analysis from a composite engineering perspective. To bridge this gap, correlating experimentally obtained results with fundamental failure mechanisms is essential for the broader application of recycled composites in real-world scenarios. One of the most promising approaches to achieving this is the use of simulation-based analysis, particularly for unidirectional fiber-reinforced composites. Computational models such as the Representative Volume Element (RVE)-based approach can provide valuable insights into mechanical performance, failure behavior, and optimization strategies [18,19,20]. Ultimately, this approach facilitates the widespread adoption of sustainable composite materials.

However, a high-fidelity model for polymer blends is still lacking in research, limiting the credibility of simulation-based approaches. Pioneering work on modeling polymer blends was conducted by Seelig and van der Giessen [21]. They developed a numerical approach for polycarbonate (PC)/acrylonitrile–butadiene–styrene (ABS) blends, a common polymer blend primarily sourced from Waste Electrical and Electronic Equipment (WEEE) recycling. The material was modeled as a two-phase system, with ABS particles dispersed in a PC matrix, considering large viscoplastic strains, softening–rehardening behavior, and plastic dilation caused by rubber particle cavitation. Later, Hund et al. [22] used the finite element method (FEM) and constitutive models to analyze the large-strain deformation and fracture behavior of PC/ABS blends, demonstrating their accuracy under complex loading conditions, which also depended on the material composition. Eddhahak and Gaudy [23] conducted an experimental study on ABS/PC blends prepared directly from WEEE waste. The phenomenological G’Sell and Jonas constitutive law was used to predict the mechanical response, with parameter identification optimized through the nonlinear Generalized Reduced Gradient algorithm. Drozdov et al. [24,25] developed a constitutive model for the viscoplastic behavior of low-density polyethylene (LDPE) and metallocene-catalyzed polypropylene (mPP) polymer blends, treating the semicrystalline polymer as a two-phase composite medium. The model was used to examine the effect of annealing on the viscoplasticity and elastic moduli of the amorphous and crystalline phases. More recently, Reuvers et al. [26] developed a thermodynamically consistent model for polyamide 6 (PA6) blended with an amorphous copolymer, incorporating nonlinear viscoelastic and elastoplastic behavior at finite strains. The mechanical and thermal properties were also characterized.

The objectives of this paper are to extend an advanced polymer model from pure polymers to blends, with the necessary considerations, covering both high-performance and commodity thermoplastics. Section 2 provides an overview of thermoplastics, polymer blends, and blend-based composites. Section 3 analyzes the stress–strain response of three representative polymer blends (polyaryl ether sulfone) (PES)/PEEK, PPS/PEEK, and HDPE/PP) and presents the rate- and temperature-dependent advanced polymer model, along with the transformation for polymer blends, considering the effects of composition on elastic modulus, viscoplasticity, and rupture. Model validation is provided through predictions of mechanical behavior across the entire weight fraction spectrum. Section 4 discusses the RVE model for simulating recycled polymer blend-based composites under critical transverse tensile loading. Section 5 presents accurate mechanical predictions and insights into the nonlinear interactions between the matrix and fibers, based on high-fidelity finite element micromechanical simulations. Additionally, it explores the feasibility of using recycled resin matrices in UD composites by comparing their performance with that of pure polymer matrices. Discussion on the guidance for incorporating polymer blends into composites is presented with the aid of an RVE simulation, and their suitability is quantified using a tentative composite performance index in Section 6.

## 2. Overview on Thermoplastics in Pure, Blended, and Composite Forms: Current Applications and Challenges

**Figure 1:**
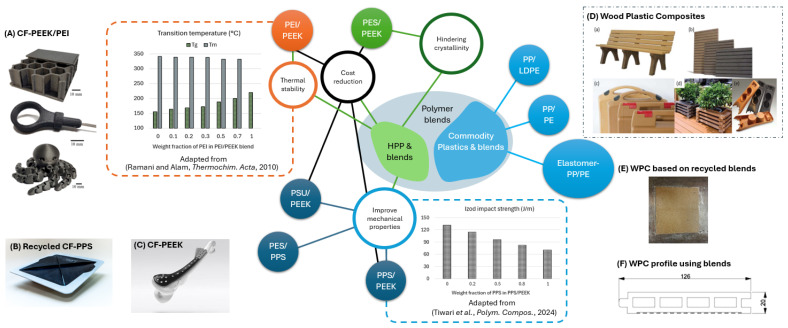
Thermoplastics and polymer blends as matrices for fiber-reinforced composites and their commercial applications in industry: (**A**) CF-PEEK/PEI used for additive manufacturing [27]; (**B**) Rotorcraft access panel from recycled carbon PPS (source: https://thermoplasticcomposites.nl/rotocraft-access-panel-from-recycled-carbon-pps/(accessed on 30 March 2025)); (**C**) Commercial CF reinforced PEEK-OPTIMA^TM^ (source: https://invibio.com/en/application-areas/trauma (accessed on 30 March 2025)); (**D**) Commercial products made from wood plastic composites (WPC) ((**a**) Park furniture; (**b**) Screening; (**c**) Tool box; (**d**) Plant boxes; (**e**) Car interior) [28]; (**E**) WPC based on the recycled PE blends from municipal waste [29]; and (**F**) WPC profile manufactured from recycled plastic blends [30].

Thermoplastics have emerged as indispensable materials across industries due to their recyclability and adaptability. These polymers are broadly categorized into high-performance polymers (HPPs) and commodity grades, each serving distinct roles in pure, blended, and composite forms, as shown in Figure 1.

### 2.1. High-Performance Thermoplastics

In their pure state, high-performance thermoplastics such as PEEK, PPS, and polyaryletherketone (PAEK) dominate applications requiring exceptional mechanical strength, thermal stability (>200 °C), and chemical resistance [2]. These materials are widely used in aerospace, automotive, and biomedical sectors for load-bearing components, such as aircraft brackets or surgical implants. Their ability to undergo repeated melting and solidification without significant degradation aligns with circular economy goals, enabling reprocessing into high-value products. However, their high cost, energy-intensive processing, and limited compatibility with additives hinder broader adoption [3].

To address these limitations, high-performance polymer blends have gained increasing attention [6,7,31,32,33], owing to their enhanced recyclability, improved thermal stability, optimized viscosity, superior mechanical performance, and better processability (see Figure 1). Ramani and Alam [32] compared the glass transition temperature ($T_{g}$) and melting temperature ($T_{m}$) of various PEEK/poly(ether imide) (PEI) blend compositions and concluded that a 50/50 PEEK/PEI ratio offers optimal thermal stability. Blends such as PEEK/PEI [27] or PPS/recycled PEEK reinforced with carbon nanotubes (CNTs) balance stiffness and toughness while improving processability [14]. These systems are increasingly utilized in high-temperature aerospace components and biomedical load-bearing applications [4]. Yet, recycling these blends remains challenging due to phase separation and difficulties in maintaining homogeneity, limiting their use in closed-loop systems [5].

When reinforced with carbon or glass fibers, high-performance thermoplastics form composites with unparalleled strength-to-weight ratios. These composites excel in structural aerospace and automotive parts, offering damage tolerance and fatigue resistance. However, recycling these composites is complicated by the need to separate fibers from the matrix, often leading to downcycling.

### 2.2. Commodity Thermoplastics

Commodity polymers such as PP, PE, and polyethylene terephthalate (PET) are ubiquitous in packaging, consumer goods, and automotive interiors due to their low cost and ease of processing [8]. Their high recyclability supports sustainable, high-volume production, although their lower thermal and mechanical performance restricts use in structural applications.

Blending commodity polymers, such as elastomer-modified PP/PE matrices, enhances properties like impact strength and modulus while integrating recycled content [11]. These blends are increasingly paired with natural fibers (e.g., wood, banana fibers) to create sustainable wood plastic composites (WPCs) for automotive interiors and construction panels [9,12,13,29,30]. Innovations in 3D printing further enable the reuse of recycled commodity blends, although inconsistent properties in recycled materials often limit performance [34].

Commodity composites, typically reinforced with natural or recycled synthetic fibers, offer lightweight, low-cost solutions for non-structural applications [13]. However, recycled thermoplastics in these composites frequently exhibit degraded thermal stability and mechanical properties due to chain scission and contamination, reducing their viability for demanding uses. Poor fiber–matrix adhesion in recycled systems further complicates performance.

### 2.3. Challenge

The integration of thermoplastics into advanced composites necessitates robust modeling to predict interactions between the constituents with complicated nonlinear behaviors. While multiscale simulations based on the RVE approach provide insights, there is a gap in understanding how this simulation approach can be used to explore the effects of using blends as matrices. In part, this is due to the current recycling barriers that persist for both categories; high-performance systems struggle with phase separation, while commodity blends face variability in recycled feedstocks. Limited data exist to evaluate the viability of recycled polymer blends for composite applications, highlighting a gap that has motivated this research. This study represents an initial exploration into the feasibility of using recycled thermoplastics as composite matrices, combining insights from the existing literature with advanced constitutive modeling to assess their performance. By integrating established material data with computational frameworks validated through experimental studies, this work aims to bridge critical knowledge gaps and establish foundational guidelines for sustainable composite design.

## 3. Blend-Based Polymer Matrix and Constitutive Modeling

This section examines the mechanical response of polymers in their pure state. As previously noted, comprehensive experimental data on polymer blends, particularly stress–strain behavior across a broad compositional range, remains limited. This study focuses on PEEK- and PP-based polymers and their respective blends. The subsequent subsections provide the detailed specifications and properties of these materials.

An advanced polymer model is first briefly introduced to characterize the representative stress–strain response of pure polymers, emphasizing the critical mechanical attributes that must be accurately captured for reliable predictive capabilities. The model is then systematically extended to polymer blends, incorporating the variability in mechanical characteristics, including elastic modulus, peak yield stress, and failure behavior, to ensure robust and generalized applicability.

### 3.1. Mechanical Response of Pure Polymers

**Figure 2:**
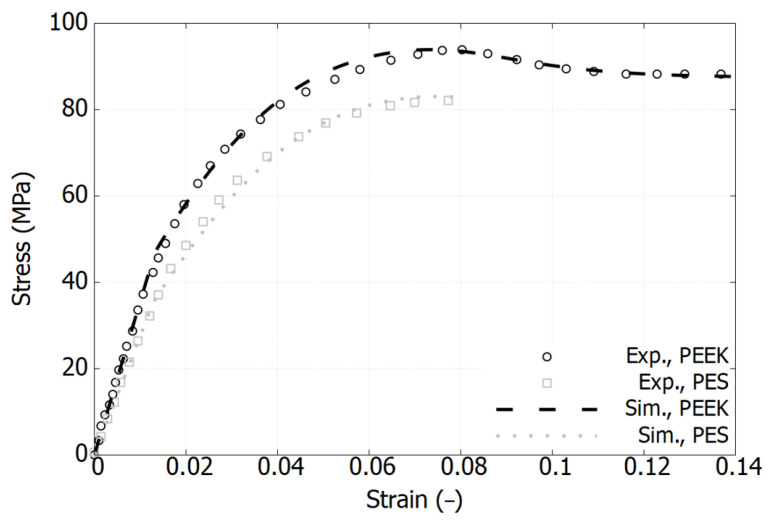
Comparison of experimental and simulation results of stress–strain curves for PES and PEEK in their pure state. Experimental data extracted from reference [31].

**Figure 3:**
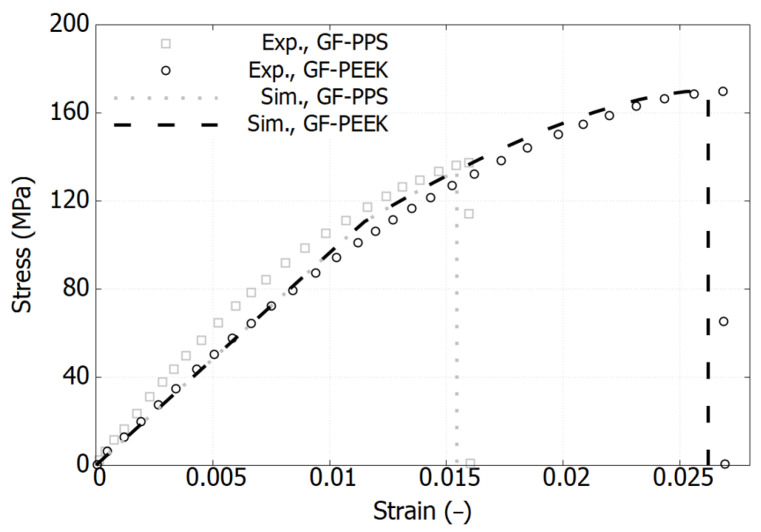
Comparison of experimental and simulation results of stress–strain curves for GF-PPS and GF-PEEK in their pure state. Experimental data extracted from reference [17].

**Figure 4:**
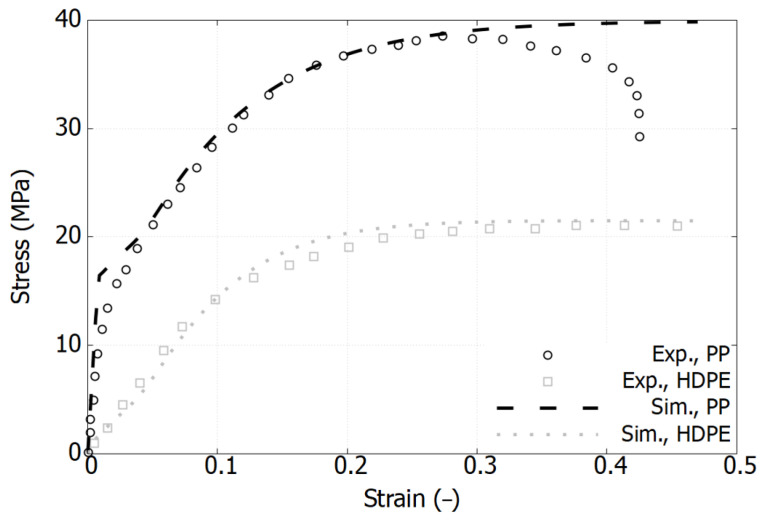
Comparison of experimental and simulation results of stress–strain curves for HDPE and PP in their pure state. Experimental data extracted from reference [11].

Figure 2, Figure 3 and Figure 4 plot the stress–strain responses of six different polymers used in the blends, categorized into the following three groups: PES and PEEK, GF-PPS and GF-PEEK, and HDPE and PP. These polymers exhibit distinct mechanical responses in terms of both elastic and plastic properties [11,17,31].

Figure 2 illustrates that both PES and PEEK exhibit similar stress–strain response trends; however, PES demonstrates a slightly lower elastic modulus and reduced peak stress compared to PEEK. Specifically, the peak stress of PES reaches 83 MPa, while that of PEEK attains 92 MPa, both occurring at a strain of 0.075. The failure strain of PES is 0.08, occurring just beyond its peak stress, whereas PEEK undergoes strain softening beyond its peak stress, ultimately failing at a strain of 0.15. Figure 3 shows that the incorporation of short glass fibers (GFs) significantly enhances the mechanical properties of commercial PPS and PEEK filled with 40% and 30% GF, respectively. Specifically, the elastic modulus exhibits a substantial increase compared to the pure polymer counterparts, exceeding 9644 MPa, as observed in the 30% GF-filled PEEK. Additionally, the ultimate strength notably improves; however, the materials exhibit increased brittleness, leading to early failure. The failure strain is 0.016 for GF-PPS and 0.027 for GF-PEEK, while in the case of GF-PPS, the material does not reach a fully saturated peak stress before failure.

The stress–strain response of the commodity polymer group is presented in Figure 4. Compared to high-performance polymers, these materials exhibit significantly lower strength levels, with HDPE reaching 21 MPa and PP attaining 38 MPa. The post-yield behavior of HDPE and PP differs considerably; HDPE exhibits a saturated stress plateau, whereas PP undergoes a sudden drop beyond a strain of 0.3, attributed to the necking effect—a geometrically induced phenomenon that was not decoupled using conventional engineering stress–strain measures. The original data for HDPE extends up to a strain of 1200%, making it challenging to extract precise data points within the elastic region, as the curves are densely clustered in a narrow range. Consequently, the elastic phase is represented based on the elastic modulus values provided in the corresponding table within the reference [11].

### 3.2. Constitutive Model for Pure Polymers

Based on these characteristics, we present our Unified Semi-Crystalline Polymer (USCP) model, which is capable of providing accurate predictions. It is important to emphasize that this study primarily focuses on the composition of polymer blends. Moreover, research on the rate sensitivity of the plastic response is scarce in the existing literature. Consequently, rate sensitivity is excluded from this work, with particular attention given to the calibration strategy employed.

**Figure 5:**
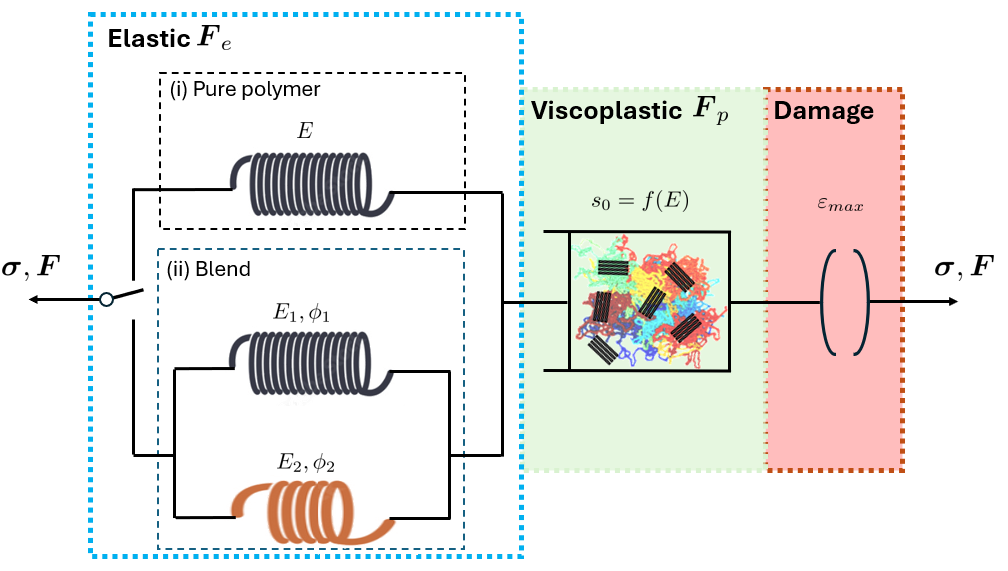
Schematic representation of elastic modulus, intermolecular resistance, and damage in corresponding rheological model.

The original USCP model [35], formulated within the finite strain kinematic framework, is a generalization of the Boyce–Parks–Argon (BPA) model [36]. It incorporates a single viscoplastic law that unifies the amorphous and crystalline phases of the polymer, providing a comprehensive approach to modeling polymer behavior. Figure 5 illustrates the rheological analogue.

The Cauchy stress tensor in the intermolecular branch σ is obtained by eliminating the plastic deformation gradient $F_{e}=FF_{p}^{-1}$, with ${\dot{F}}_{p}=F_{e}^{-1}{\tilde{D}}^{p}F_{e}F_{p}$. The rate of inelastic deformation is written as

(1) $${\tilde{D}}^{p}=\dot{\bar{\varepsilon }}\;N,$$

where N is the direction tensor, and $\dot{\bar{\varepsilon }}$ is the effective plastic strain rate.

(2) $$\dot{\bar{\varepsilon }}={\dot{\varepsilon }}_{0}\exp\left[-\frac{A\left(s-\alpha \sigma_{h}\right)}{\theta }\left(1-{\left(\frac{\sigma_{\mathrm{eq}}}{s-\alpha \sigma_{h}}\right)}^{m}\right)\right],$$

where $\sigma_{\mathrm{eq}}$ is the defined equivalent stress. The hydrostatic part of the Cauchy stress tensor is $\sigma_{h}$, and the description of model parameters are given in Table A1. The evolution law for the athermal effective stress is formulated using a smooth, Heaviside-like function to capture the pre-peak hardening, post-peak softening, and second yield behavior resulting from the crystalline phase contribution, as follows:

(3) $$\begin{array}{c} \dot{s}=H_{1}\left(\bar{\varepsilon }\right)\cdot \left(1-\frac{s}{s_{1}}\right)\cdot \dot{\bar{\varepsilon }}+H_{2}\left(\bar{\varepsilon }\right)\cdot \left(1-\frac{s}{s_{2}}\right)\cdot \dot{\bar{\varepsilon }}+H_{3}\left(\bar{\varepsilon }\right)\cdot \left(1-\frac{s}{s_{3}}\right)\cdot \dot{\bar{\varepsilon }},\\ \;\mathrm{with}\;s_{0}=\sqrt{3}\frac{8.5^{-1/m}}{\left(1-\nu \right)}\frac{E}{2(1+\nu )} \end{array}$$

where the athermal strength $s_{i}$ (i = 1, 2, 3) corresponds to the preferred states at different stages of deformation, as described in reference [35]. The functions governing the hardening evolution are expressed as follows:

(4) $$H_{1}\left(\bar{\varepsilon }\right)=-h_{1}\left\{\tanh\left(\frac{\bar{\varepsilon }-{\bar{\varepsilon }}_{p}}{f{\bar{\varepsilon }}_{p}}\right)-1\right\},$$

(5) $$H_{2}\left(\bar{\varepsilon }\right)=h_{2}\left\{-\tanh\left(\frac{\bar{\varepsilon }-{\bar{\varepsilon }}_{p}}{f{\bar{\varepsilon }}_{p}}\right)\tanh\left(\frac{\bar{\varepsilon }-{\bar{\varepsilon }}_{c}}{f{\bar{\varepsilon }}_{c}}\right)+1\right\},$$

(6) $$H_{3}\left(\bar{\varepsilon }\right)=h_{3}\left\{\tanh\left(\frac{\bar{\varepsilon }-{\bar{\varepsilon }}_{c}}{f{\bar{\varepsilon }}_{c}}\right)+1\right\},$$

where ${\bar{\varepsilon }}_{p}$ is the plastic strain at the peak yielding point, and ${\bar{\varepsilon }}_{c}$ is the characteristic plastic strain when the crystalline nano-block initiates the yielding process. This formulation involves three hardening (or softening) parameters $h_{1}$, $h_{2}$, and $h_{3}$. The parameter *f* is the smoothing factor.

As shown in Figure 2, Figure 3 and Figure 4, the model accurately captures the behavior and has been validated for pure polymers. It is now extended to polymer blends.

### 3.3. Extension Version for Blends

#### 3.3.1. Elastic Modulus

It is intuitive to incorporate the mix ratio-dependent elastic modulus into the model (see Figure 5). PES was procured from Amoco Performance Products under the trade name Radel (grade A-300), while PEEK was synthesized in the laboratory as described by [37]. The HDPE used was HD 6605, a homopolymer with a melt flow index of 5 g/10 min (ExxonMobil Chemical), and the PP was Certene PHM-20AN, a homopolymer with a melt flow index of 20 g/10 min (Muehlstein and Co., Inc.) [11]. Both PES/PEEK [37] and HDPE/PP [11] systems followed the ASTM D 638 method [38] for conducting the tensile test.

The blend moduli were reported to exhibit a composition-dependent behavior, as described by the general equation proposed by Nielsen and Landel [39] for one-phase binary mixtures, and further specified by the form presented by Kleiner [40], as follows:

(7) $$E=E_{1}\phi_{1}+E_{2}\phi_{2}+\beta_{12}\phi_{1}\phi_{2}$$

where $\beta_{12}$ is the term that represents the magnitude of the deviation from nonlinearity, with the best-fitted values being 1000 for the PES/PEEK system and 50 for the HDPE/PP system. Variable $\phi_{i}$ (i=1,2) denotes the volume fraction of each component, where $\phi_{1}$ can be calculated and converted from the given weight fraction $w_{1}$ of one component using the following equation:

(8) $$\phi_{1}=\frac{\rho_{2}}{\rho_{1}\left(1/w_{1}-1\right)+\rho_{2}}$$

where the density values are sourced from the literature and the corresponding datasheets of the commercial products, as provided in Table 1.

**Figure 6:**
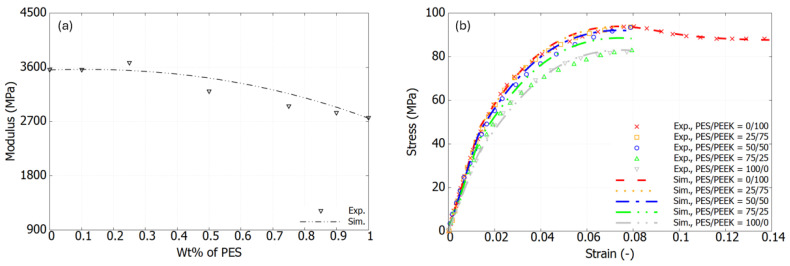
PES/PEEK blends: (**a**) Experimental data and best-fit values of elastic modulus; (**b**) Comparison between experimental and simulation results of stress–strain response.

**Figure 7:**
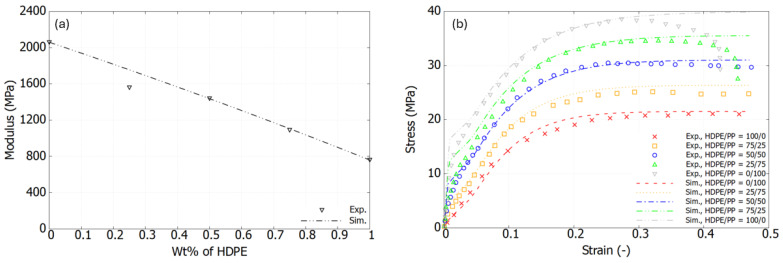
HDPE/PP blends: (**a**) Experimental data and best-fit values of elastic modulus; (**b**) Comparison between experimental and simulation results of stress–strain response.

Figure 6a and Figure 7a show good agreement between the experimental results and the calculated ones. According to Equation (3), the composition dependency is naturally incorporated into the plastic formulation. For instance, Figure 6b shows a comparison between the experimental and simulation results using the extended version of the USCP model for the PES/PEEK blends. The stress–strain curves for various mix ratios are well captured, with the nonlinear portion following a smooth trend, reaching the peak yield point consistently. Shown in Figure 7b, good agreement is observed between the experimental results and the simulation for the HDPE/PP blends.

#### 3.3.2. Failure

Figure 5 shows that the proposed model is constructed based on multiple modules. To characterize failure, a damage module is introduced. The model’s capability is demonstrated using a failure criterion based on the maximum failure strain, in accordance with the experimental observations of brittle fracture. Certainly, various energy-based damage theories can also be incorporated into the model.

Previous research by Tiwari et al. [17] investigated the mechanical behavior of PPS, PEEK, and their blends. PPS, a semi-crystalline polymer with a melting temperature of 279.68 °C, was studied in its reinforced form. Specifically, they examined 40% short glass fiber (GF)-filled PPS (Ryton®-R-4-230 NA) from Solvay and 30% short GF-filled PEEK (PEEK 450 GL 30, Victrex, Lancashire, UK). In this work, the USCP was adopted directly to the GF-filled thermoplastics, and the failure strain of blends were considered using the rule of mixture, as expressed in the following equation:

(9) $$\varepsilon_{f}=\varepsilon_{f1}\phi_{1}+\varepsilon_{f2}\phi_{2}$$

**Figure 8:**
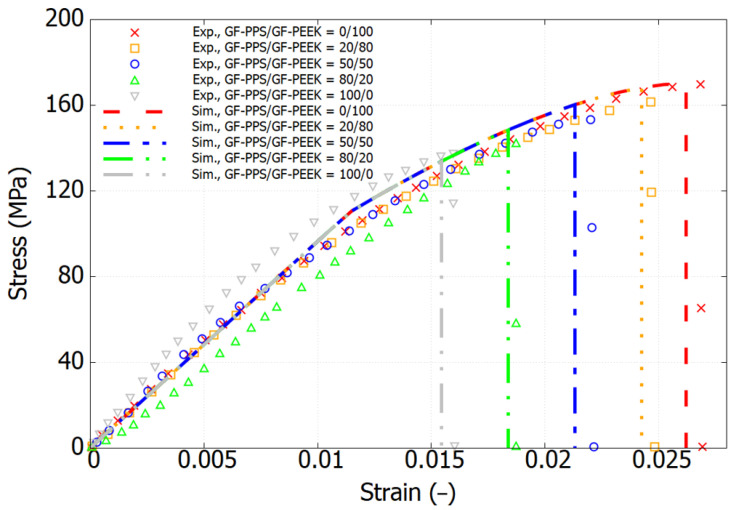
Experimental and simulation results of stress–strain curves for GF-PPS/GF-PEEK matrix.

Figure 8 presents the stress–strain response for various mix ratios of GF-PPS and GF-PEEK. The simulation results accurately capture the overall trend, with the predicted failure showing strong agreement with the experimental data.

We demonstrate that our model provides reliable predictions, and it is subsequently used as the matrix in the RVE simulations.

## 4. High-Fidelity FE Micromechanical RVE Model

To investigate the influence of matrix blend composition on the micromechanical behavior of a unidirectional (UD) composite ply, the development of an RVE model is crucial. Constructing this RVE involves defining the geometry and boundary conditions, as well as selecting the appropriate material constitutive models.

**Figure 9:**
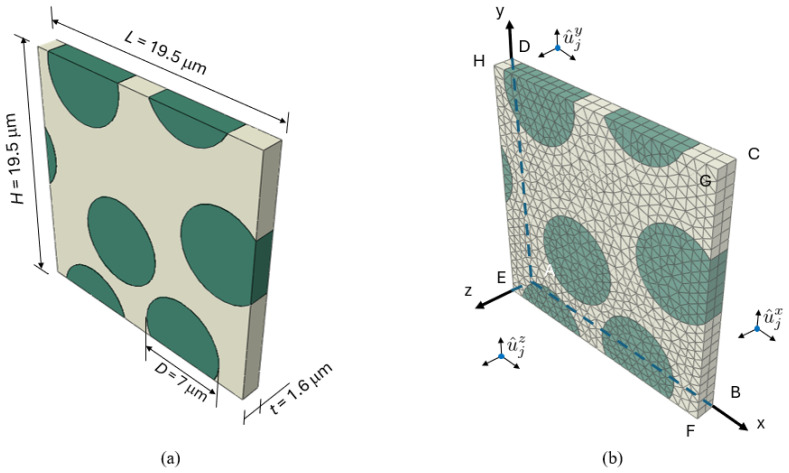
(**a**) Representative Volume Element (RVE) model and (**b**) node-coupling implementation of MPBCs.

For this study, the chosen fiber material is UD carbon with a diameter of 7 μm, represented by randomly positioned cylinders embedded in a polymer matrix under periodic boundary conditions. Figure 9 illustrates the geometry of the model, which contains four fibers with a fiber volume fraction of 40%. The fiber positions are generated using a random algorithm previously applied in the molecular dynamics simulations of two-dimensional cohesive granular materials [41].

Both the fibers and matrix are independently meshed using three-dimensional (3D) six-node wedge elements from the element library, designated as C3D6 [42]. The element size is carefully analyzed to be sufficiently small, approximately 0.8 μm, ensuring the accurate resolution of microstructural features and consistency in the results. Mechanical Periodic Boundary Conditions (MPBCs) are implemented using the node-to-node coupling approach proposed by Garoz et al. [19]. These MPBCs enable the application of a mechanical load in the transverse direction while allowing Poisson’s contraction.

It is assumed that the carbon fibers follow a linear elastic and transversely isotropic response. Properties of the mechanical and thermal constitutive behaviors are adopted from Arteiro et al. [18]. The properties are presented in Table 2. The fiber–matrix interface is modeled using surface-to-surface cohesive contact as an alternative to surface-based tie constraints. In this approach, there is no need to specify the stiffness or damage properties of the cohesive contact behavior.

## 5. Numerical Analysis of UD Composite Using Blend-Based Matrices

**Figure 10:**
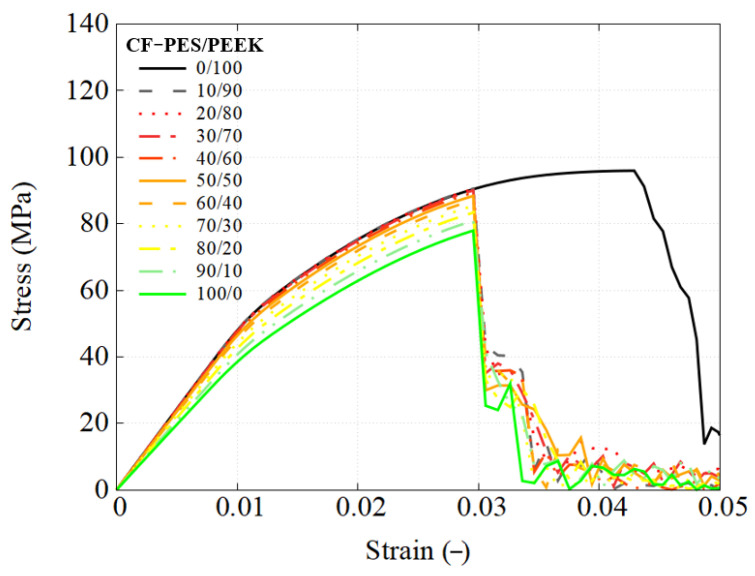
Engineering stress–strain response of CF-PES/PEEK composites using RVE simulation.

**Figure 11:**
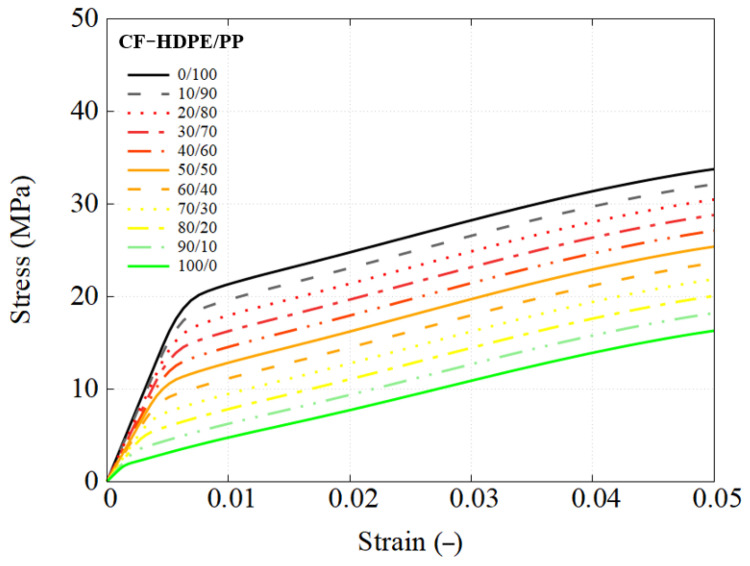
Engineering stress–strain response of CF-HDPE/PP composites using RVE simulation.

**Figure 12:**
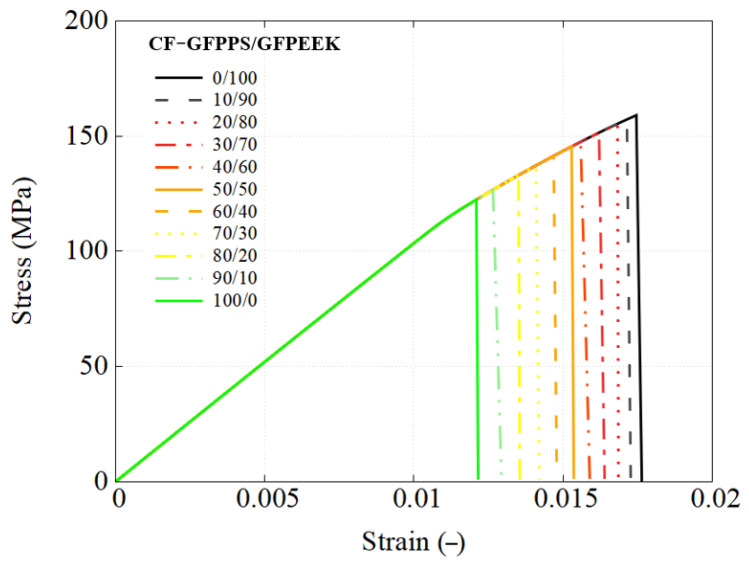
Engineering stress–strain response of CF-GF-PPS/GF-PEEK composites using RVE simulation.

This section details the performance response of UD composites using the previously developed RVE model, with an attempt to utilize different types of blends. The stress–strain response is straightforwardly obtained using engineering measures, where stress is calculated from the applied load and strain is the nominal strain. The predicted mechanical response of composites using each blend used as the matrix are presented in Figure 10, Figure 11 and Figure 12.

**Figure 13:**
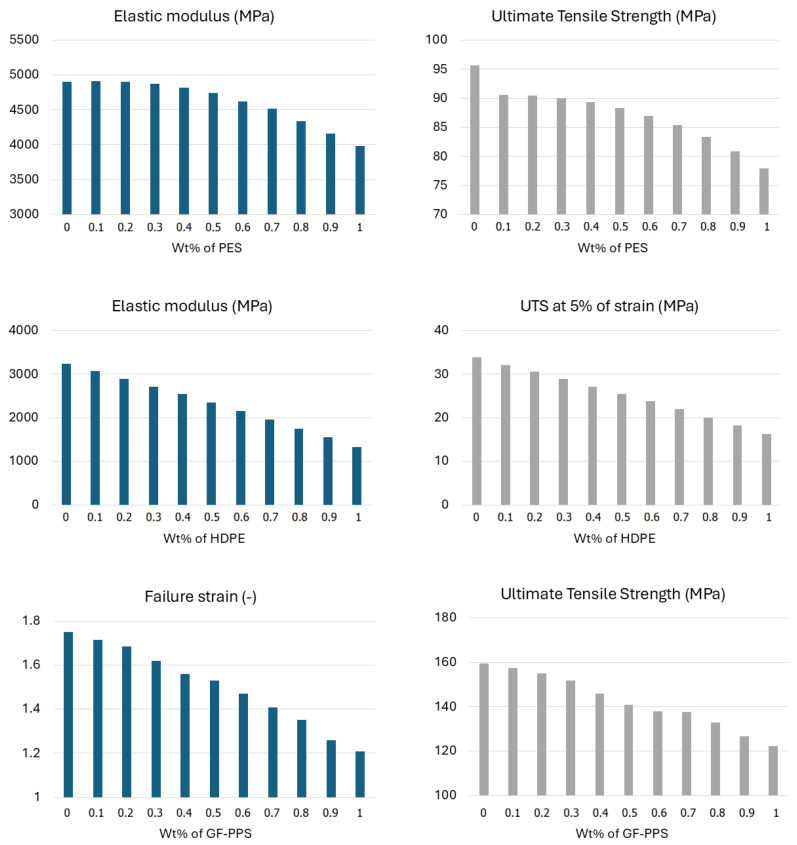
Summary of mechanical properties from RVE simulations for three different polymer blend composite systems.

The stress–strain behaviors of all the composites closely follow those of their corresponding blends. The incorporation of carbon fibers significantly enhances the overall mechanical performance, resulting in increased stiffness and strength. The key mechanical properties are summarized in Figure 13.

However, the addition of carbon fibers also introduces brittleness, leading to a substantial reduction in failure strain. For example, the PES/PEEK matrices in Figure 6 exhibit a failure strain of approximately 8%, whereas their composite counterparts (Figure 10) fail at around 3%. This reduction occurs because localized matrix pockets within the RVE of the composite reach their failure strain before the overall composite fails at the global scale.

Similarly, for the CF-GF-PPS/GF-PEEK composites, the failure strain ranges from 1.2% to 1.75%, while localized matrix regions experience failure at an even earlier stage. This phenomenon is further examined through fiber–matrix interaction investigation and load transfer analyses, supported by detailed results of RVE simulations. The findings are accompanied by corresponding stress–strain curves, visualizations of stress and strain distributions, and plasticity indicators to provide deeper insights into the composite failure mechanisms.

A comparative analysis of the RVE performance using pure polymers and selected 50/50 blends is conducted across three blend groups, highlighting performance differences and the effectiveness of recycled matrices in maintaining UD composite integrity.

**Figure 14:**
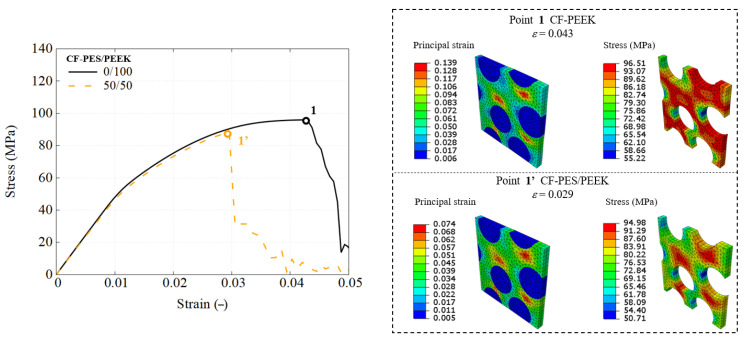
Global and local mechanical responses of CF-PES/PEEK composites, as demonstrated through RVE simulation.

Figure 14 compares the following two different matrix systems: pure CF-PEEK as the baseline material and a 50/50 PES/PEEK blend. The global engineering stress–strain curves reveal minimal differences between the two, with a UTS of 95.7 MPa for the PEEK-based composite and 88.35 MPa for the PES-modified PEEK matrix. Strain and stress distributions are analyzed at critical loading points where matrix failure occurs. Both principal strain profiles exhibit similar failure locations but different strain values, influenced by fiber clustering effects. At global failure, the pure PEEK-based composite reaches a maximum strain of 13.9%, while the PES-modified composite fails at 7.4%. The peak matrix stress aligns with the UTS in Figure 6, indicating that incorporating 50 wt% PES effectively reduces the reliance on costly PEEK while maintaining mechanical performance, with only a minor reduction in failure strain of less than 1.5%.

**Figure 15:**
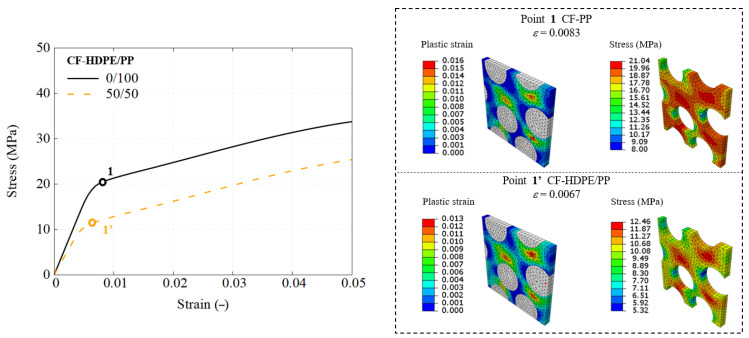
Global and local mechanical responses of CF-HDPE/PP composites, as demonstrated through RVE simulation.

**Figure 16:**
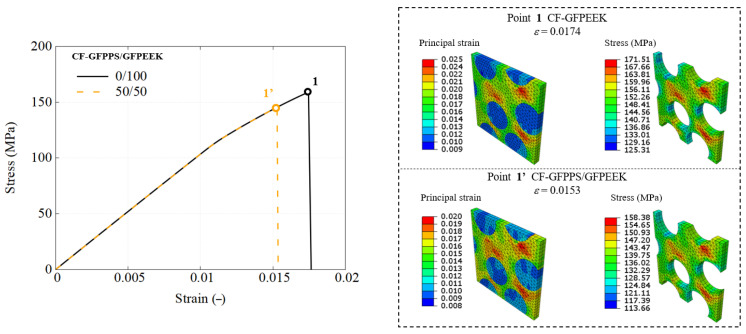
Global and local mechanical responses of CF-GFPPS/GFPEEK composites, as demonstrated through RVE simulation.

Figure 15 and Figure 16 demonstrate that the observations made for the PES/PEEK system are also applicable to the other two polymer blend systems. However, in the CF-HDPE/PP system, no failure was observed in the FEM simulations, so the chosen loading stage corresponds to the yielding point.

## 6. Discussion

The RVE simulations suggest that incorporating recycled polymer blend matrices into UD composites is a feasible approach, despite a few trade-offs. The obtained stress–strain curves indicate that while the addition of carbon fibers enhances overall stiffness and strength, it also leads to a reduction in failure strain compared to pure polymer systems. As mentioned, in the PES/PEEK system, the failure strain drops from approximately 8% in the neat blend to around 3% in the composite, primarily due to localized matrix failure. Despite this decrease in ductility, the prospect of reducing material costs and enhancing sustainability (by partially replacing expensive, high-performance polymers like PEEK with recycled materials) presents a compelling case for these blends in applications where extreme ductility is not critical.

The trade-off between performance and sustainability is a central consideration. Although thermoplastics in pure form may offer better ductility, recycled blends maintain comparable strength and stiffness, and their use could still bring lower costs and better environmental impact. When viewed from a life-cycle and sustainability perspective, the slight reduction in mechanical performance is often an acceptable compromise, especially in applications where maximum ductility is not the overriding requirement.

The simulations also suggest that the optimal usability of these blends depends on the operating conditions. They are best suited for environments demanding high load-bearing capacity under controlled loading conditions, such as those involving transverse tensile loads. It is critical, however, to account for the reduced failure strain and to optimize the microstructure by getting a more homogeneous fiber distribution. This may help mitigate early-stage failure due to localized stress concentrations.

To quantify the suitability of each blend, a tentative Composite Performance Index (CPI) could be formally introduced as follows:

(10) $$CPI=\alpha \left(\frac{UTS}{{UTS}_{pure}}\right)+\beta \left(\frac{\varepsilon_{f}}{\varepsilon_{f,pure}}\right)+\gamma \;SF\;,$$

where the first terms between the parenthesis represents the normalized ultimate tensile strength, which reflects the load-bearing capacity of the composite. The second term is the normalized failure strain that indicates the relative level of ductility when using blends in the composite. The last one corresponds to a sustainability factor SF that might account for the cost reduction and environmental benefits of using recycled polymers, among other advantages. The coefficients α,β,γ are weights determined by the application requirements. With respect to SP, a tentative way to quantify this factor is to break it down into normalized, dimensionless components in which each one represents a key aspect or, more formally, the following:

(11) $$SF=\sum_{k}\;c_{k}\;S_{k}\;\mathrm{with}\;S_{k}=1-\frac{\chi_{k,\mathrm{blend}}}{\chi_{k,\mathrm{pure}}}\;,$$

where $S_{k}$ is the relevant component to assess sustainability, and $c_{k}$ are weighting coefficients that reflect the relative importance assigned to each component, fulfilling the condition $\sum \;c_{k}=1$. Three plausible components to assess SF might be $S_{\mathrm{env}}$ that can quantify any relevant environmental impact metrics (e.g., ${\mathrm{CO}}_{2}$ emissions) of the recycled and pure materials, $S_{\mathrm{cst}}$ for the cost of recycled blends and pure materials, and $S_{\mathrm{rsc}}$ could indicate the reduction in virgin material consumption when using recycled polymers. According to this proposal, as an example, for k=cst, the descriptors $\chi_{\mathrm{cst},\mathrm{blend}}$ and $\chi_{\mathrm{cst},\mathrm{pure}}$ can be assigned to the cost of the recycled blend and pure polymers, respectively. It is worth mentioning that by adjusting the weights $c_{k}$, the industrial agent can tailor the index to reflect the specific priorities of a given application or product, whether they be environmental impact, cost savings, or resource conservation.

In terms of methodology, to ensure the effective implementation of the proposed CPI across industries, the following adaptation and customization guidelines are proposed: First, industries must define clear objectives by identifying primary application requirements, such as mechanical performance (e.g., strength, ductility), cost constraints, and environmental impact thresholds, to establish a baseline for index alignment. Next, the appropriate weights should be assigned to the coefficients of the Index to ensure that priorities are transparently reflected; for instance, sectors like packaging, governed by stringent EU sustainability mandates, might prioritize environmental impact over short-term cost savings. Third, the relevant sustainability indicators—such as ${\mathrm{CO}}_{2}$ reduction metrics ($S_{\mathrm{env}}$), waste reducibility targets ($S_{\mathrm{rcs}}$), or cost-effectiveness ratios ($S_{\mathrm{cst}}$)—should be selected, and they must align with the industry-specific goals, ensuring measurable and actionable outcomes. Finally, contextual calibration through industry-specific benchmarking is critical to validate the accuracy of the index, leveraging empirical data (e.g., Packaging and Packaging Waste Regulation (PPWR) [43] compliance thresholds or Plastics Recyclers Europe (PRE) [44] emission estimates) to refine its components. This structured approach not only bridges theoretical frameworks with practical industrial needs but also empowers sectors to balance performance, sustainability, and economic viability in a quantifiable and adaptable manner.

On the other hand, simulations point out that the early onset of localized failure, particularly under high strain, represents a key limitation. This shortfall may restrict the use of recycled blends in applications that require both high strength and high ductility, though they remain attractive for applications where the highest level of ductility is not essential. In that sense, it is important to note that some of these limitations might be associated with the assumptions inherent in the simulations. The RVE model assumes ideal fiber–matrix bonding and a perfectly homogeneous matrix (no defects like voids), factors that might lead to an overestimation of composite performance relative to real-world production. A fundamental aspect of the recycling process, it significantly influences the final mechanical performance of the resulting material. In this study, our focus begins at the stage where recycled materials have already been produced and are ready for use, similar to the characterization of other pristine materials. However, a broader quantification of recycling effects, such as changes in molecular weight, impurity content, or crystallinity, is not included. Consequently, discrepancies between the simulated and experimental results are expected, and these assumptions underscore the need for further experimental validation to refine the predictive models.

Nevertheless, the prospects for additional blend formulations and innovative composite architectures appear promising. Experimenting with different blend ratios, incorporating secondary additives or nanofillers, and exploring hybrid composite designs that combine recycled and conventional matrices can potentially overcome current limitations. Moreover, adjusting the fiber orientations and the stacking sequences in UD-based laminates may help mitigate localized failures and further enhance mechanical performance.

## 7. Conclusions

In this work, an advanced constitutive model originally developed for pure thermoplastic polymers was successfully extended to capture the elasto-viscoplastic behavior of polymer blends, specifically demonstrating its applicability to both high-performance (PEEK-based) and commodity (PP-based) polymer systems. The model accurately accounts for composition-dependent variations in mechanical properties, such as elastic modulus and strength, and includes a damage module employing a strain-based failure criterion, effectively characterizing material failure.

Using Representative Volume Element (RVE)-based finite element simulations, the developed model provided local and global predictions of the mechanical performance under critical transverse tensile loading conditions. These simulations showed that the stress–strain behaviors of composite materials that incorporated recycled thermoplastic blends as matrices closely aligns with those of their corresponding neat polymer blends. However, the simulations also revealed that employing recycled blends as a matrix involved a reduction in ductility. It was observed that localized strain concentrations caused premature matrix cracking at the microscale, particularly in recycled blend matrix-rich regions between fibers.

The approach outlined here aims to minimize the reliance on extensive trial-and-error by providing a computational framework for evaluating recycled polymer blends in industrial composite applications. This method could lead to significant reductions in material costs, particularly when substituting costly polymers like PEEK, without compromising mechanical performance.

To evaluate the feasibility of using recycled polymer blends in composites, this study introduces a performance index that integrates both mechanical and sustainability metrics. Mechanical metrics, specifically the normalized ultimate tensile strength and normalized failure strain, are derived directly from the RVE simulation results. Although a detailed quantitative sustainability assessment remains an area for future investigation, the study proposes a preliminary structure for a sustainability factor. This factor can be effectively combined with mechanical metrics to enable a comprehensive evaluation of blend-based composites. Overall, the suggested performance index provides a clear, structured methodology to quantitatively determine the suitability of recycled polymer blends for targeted composite applications.

While the studied blends may exhibit reduced ductility relative to the pure thermoplastics, their comparable strength and the environmental benefits might represent attractive trade-offs for many industrial scenarios. However, careful consideration of the operating conditions and loading scenarios remains essential to fully exploit the advantages offered by recycled blend matrices. Future efforts should focus on refining the sustainability quantification indicators.

On the other hand, it is worth mentioning that this numerical investigation highlighted several aspects that require further exploration. Future work should address experimental validation under various loading conditions to refine model predictions, particularly addressing realistic fiber–matrix interface features. Additionally, accurately capturing and reconstructing realistic microstructures using advanced microscopy techniques such as SEM—including the presence of voids, heterogeneities, and variability in fiber distribution—is essential for enhancing the fidelity and predictive capability of models, going beyond the information provided by conventional stress–strain curves.

Nevertheless, this study provides valuable initial insights into the mechanical viability and sustainability advantages of using recycled thermoplastic blends as matrices for unidirectional fiber-reinforced composites. Continued research efforts in model refinement, experimental validation, and microstructural optimization are recommended to fully leverage the economic and environmental benefits of these innovative composite materials.

## Figures and Tables

**Table 1:** Material properties for the investigated materials in their pure states [11,17,31].

Property	PES	PEEK	GF-PPS	GF-PEEK	HDPE	PP
ρ (g/${\mathrm{cm}}^{3}$)	1.370	1.263	1.68	1.51	0.948	0.905
*E* (MPa)	2755 *	3556 *	9644 *	9644 *	760	2060

* values obtained by extracting from the stress-strain curves.

**Table 2:** Transversely isotropic elastic properties [18] of carbon fiber.

Density	Mechanical				
ρ (kg/$m^{3}$)	$E_{11}$ (GPa)	$E_{22}$ (GPa)	$\nu_{12}$	$G_{12}$ (GPa)	$G_{23}$ (GPa)
1800	276	15	0.2	15	7

## Data Availability

Data are contained within the article.

## Appendix A. Thermomechanical Properties and Identified Model Parameters

The appendix summarizes the input parameters for the thermomechanical analyses of UD composites. Tables are provided separately for each constituent, including elasto-viscoplastic response of matrices, elastic response of carbon fiber and the traction-separation law of fiber–matrix interface. Readers are referred to the calibrated parameters for USCP model in Table A1 with the corresponding thermal properties for the investigated polymers used in this study.

**Table A1:** Material parameters for the investigated PES/PEEK [17], PPS/PEEK [31] and HDPE/PP [11].

Material Parameter	Unit	PES/PEEK	PPS/PEEK	HDPE/PP	Description
$E_{\mathrm{ref}}$	MPa	3556	9644	2060	Modulus of base polymer
ν	-	0.40	0.40	0.43	Poisson’s ratio
α	-	0	0	0	Pressure sensitivity
${\dot{\bar{\varepsilon }}}_{0}$	1/s	3266.36	3.4 × $10^{26}$	1.049 × $10^{12}$	Rate sensitivity
*m*	-	0.50	0.50	0.50	Rate sensitivity
*A*	K/MPa	1347	1272	1027	Rate sensitivity
$s_{0}$	MPa	50.74	137.6	2.29	Initial equivalent strength
$s_{1}$	MPa	98.61	210	5.1	Athermal peak strength
$s_{2}$	MPa	98.61	185.6	11.2	First saturation strength
$s_{3}$	MPa	93.02	184	27	Second saturation strength
$h_{1}$	MPa	1950	26,619	50.36	Pre-peak hardening
$h_{2}$	MPa	1837	32,546	339.6	Post-peak softening
$h_{3}$	MPa	160	160	340	Second yield hardening
${\bar{\varepsilon }}_{p}$	-	0.052	0.0088	0.0012	Peak plastic strain
${\bar{\varepsilon }}_{c}$	-	0.254	0.0188	0.0319	Activation plastic strain
*f*	-	0.3	0.3	0.3	Smooth factor
